# Genetic characterization and development of novel microsatellite markers for *Conogethes punctiferalis* (Lepidoptera: Crambidae) populations in the Republic of Korea

**DOI:** 10.1093/jisesa/ieag124

**Published:** 2026-11-14

**Authors:** Il Nam, Dong-Yeol Lee, Yujeong Park, Sanghyeon Kim, Min-Jung Huh, Min-Woo Lee, Jae-Woo Lee, Ji-Hwan Han, Seon-Woo Bang, Makoto Kobayashi, Il-Kwon Park

**Affiliations:** Pests and Weeds Control Division, Department of Agro-food Safety and Crop Protection, National Institute of Agricultural Sciences, RDA, Wanju, Republic of Korea; Department of Agriculture, Forestry and Bioresources, College of Agriculture and Life Sciences, Seoul National University, Seoul, Republic of Korea; Entomology Program, Department of Agricultural Biotechnology, Seoul National University, Seoul, Republic of Korea; Emerging Pathogens Institute and Department of Entomology and Nematology, University of Florida, Gainesville, FL, USA; College of Tropical Agriculture and Human Resources, University of Hawaii at Manoa, Honolulu, HI, USA; Department of Agriculture, Forestry and Bioresources, College of Agriculture and Life Sciences, Seoul National University, Seoul, Republic of Korea; Department of Agriculture, Forestry and Bioresources, College of Agriculture and Life Sciences, Seoul National University, Seoul, Republic of Korea; Department of Agriculture, Forestry and Bioresources, College of Agriculture and Life Sciences, Seoul National University, Seoul, Republic of Korea; Department of Agriculture, Forestry and Bioresources, College of Agriculture and Life Sciences, Seoul National University, Seoul, Republic of Korea; Uryu Experimental Forest, Field Science Center for Northern Biosphere, Hokkaido University, Hokkaido, Japan; Department of Agriculture, Forestry and Bioresources, College of Agriculture and Life Sciences, Seoul National University, Seoul, Republic of Korea; Research Institute of Agriculture and Life Science, College of Agriculture and Life Sciences, Seoul National University, Seoul, Republic of Korea

**Keywords:** yellow peach moth, transcriptome-derived microsatellite marker development, population genetic structure

## Abstract

*Conogethes punctiferalis* (Guenée, 1854) (Lepidoptera: Crambidae) is a polyphagous pest of agricultural and forest crops, but the genetic identity and fine-scale population structure of Korean populations remain poorly resolved. We combined mitochondrial cytochrome c oxidase subunit I (COI) sequence analysis with the development and application of transcriptome-derived microsatellite markers to assess lineage identity and regional population structure. Korean sequences were analyzed together with reference sequences from China and Japan using phylogenetic reconstruction, COI-based molecular operational taxonomic unit delimitation, and haplotype-network analyses. We also screened 40 candidate microsatellite loci from a larval transcriptome, retained 10 polymorphic loci, and genotyped 326 individuals from 12 Korean populations and 1 Japanese population. Korean individuals were consistently associated with the East Asian COI lineage corresponding to *C. punctiferalis* sensu stricto in the present reference dataset. Across the full 13-population dataset, microsatellite analyses showed genetic differentiation in most pairwise population comparisons alongside extensive individual-level admixture, whereas divMigrate analysis of the 12 Korean populations indicated asymmetric relative connectivity among several population pairs. These loci provide transcriptome-derived nuclear markers for further evaluation of genetic diversity, fine-scale population structure, and temporal genetic change across geographic populations.

## Introduction

*Conogethes punctiferalis* (Guenée, 1854) (Lepidoptera: Crambidae) is a polyphagous moth pest widely distributed across Asia and Oceania, where it attacks various agricultural and forest-related crops, including peach, apple, chestnut, and maize (Rojas-Sandoval 2023, Ramzan et al. 2024). The larvae bore into fruits, shoots, stems, or reproductive tissues, making early detection of infestation difficult and causing not only direct feeding damage but also reduced marketability and secondary decay (Inoue and Yamanaka 2006, Rojas-Sandoval 2023). Owing to its broad host range, wide distribution, and economic importance, *C. punctiferalis* has been the subject of numerous studies on pest management, host use, behavioral ecology, and genetic variation (Du et al. 2016). Ecological and genetic differences associated with host plants, geographic distribution, or regional populations have also been reported (An et al. 2024).

Such ecological and genetic variation is closely associated with the taxonomic complexity of *C. punctiferalis*. Indeed, nominal *C. punctiferalis* represents a taxonomically complex taxon in which host-associated and geographically differentiated lineages have been reported. Within the *Conogethes* complex, populations previously regarded as ecotypes or regional variants of *C. punctiferalis* have subsequently been reclassified as distinct species based on morphological, ecological, and molecular evidence (Honda 1986, Honda and Mitsuhashi 1989, Inoue and Yamanaka 2006, Shashank et al. 2018, An et al. 2024). For example, the Pinaceae-feeding type in Japan was formerly considered an ecotype of *C. punctiferalis* but was later reclassified as *Conogethes pinicolalis*, a distinction further supported by morphological, ecological, and molecular evidence (Inoue and Yamanaka 2006, Jeong et al. 2021). Similarly, the cardamom-feeding lineage from India was subsequently described as *Conogethes sahyadriensis* (Shashank et al. 2018). More recently, molecular evaluations have suggested deep mitochondrial divergence and the possible presence of cryptic species within *Conogethes*, including nominal *C. punctiferalis* (An et al. 2024), highlighting the need to clarify the genetic identity of regional populations and their placement within the existing *C. punctiferalis* lineage.

Effective sex pheromone blends have been reported to differ among Japanese, Chinese, and Korean *C. punctiferalis* populations (Konno et al. 1982, Liu et al. 1994, Nam et al. 2022). These reports provide behavioral context for comparing the genetic identity and population structure of East Asian populations. Whether variation in pheromone traits is associated with genetic differentiation remains to be tested.

In addition to evaluating lineage-level relationships, it is also necessary to assess the extent to which regional populations within the same genetic lineage are genetically continuous or exhibit fine-scale differentiation. This represents a fine-scale population genetic question distinct from intercountry lineage-level comparisons and can provide baseline information for understanding genetic continuity and regional differentiation among Korean populations, as well as for interpreting potential ecological and behavioral variation among regional populations. Microsatellite markers, or simple sequence repeats (SSRs), are particularly suitable for this purpose because their high polymorphism and codominant inheritance make them useful for evaluating genetic diversity, population structure, and gene flow. They have also served as informative nuclear markers for estimating regional population differentiation and dispersal connectivity (Jarne and Lagoda 1996, Sunnucks 2000, Zhang and Hewitt 2003, Selkoe and Toonen 2006). More broadly, molecular marker systems, including SSRs, have been widely applied to population genetic analyses and pest population monitoring in diverse insect taxa (Behura 2006). However, previous population genetic studies of *C. punctiferalis* have mainly relied on mitochondrial sequences or ISSR markers, and few SSR markers have been available for assessing fine-scale genetic structure and recent connectivity among its regional populations (Zhang et al. 2010, Aoshima et al. 2020, Dong et al. 2023). Although genome-wide SNP data have been generated for Chinese *C. punctiferalis* populations, regional populations in Korea remain less thoroughly characterized using multiple nuclear loci (Gao et al. 2023). Accordingly, we developed a multiplex SSR panel to assess nuclear genetic variation among Korean populations and to establish a consistent set of loci for later geographic and temporal comparisons. This targeted approach complements genome-wide SNP analyses, which remain useful when broader genomic resolution is required (Lemopoulos et al. 2019, Petermann et al. 2021).

In this study, we performed mitochondrial cytochrome c oxidase subunit I (COI) sequence analysis and population genetic analysis using novel SSR markers to evaluate the genetic identity and regional population structure of *C. punctiferalis*. First, COI sequences were used to determine the genetic placement of Korean populations within the existing *C. punctiferalis* lineage and to examine their lineage-level relationships with Japanese and Chinese populations. We then developed polymorphic SSR markers from a *C. punctiferalis* transcriptome and applied them to Korean regional populations to assess genetic diversity, population structure, and genetic connectivity. Together, these analyses provide complementary insights into lineage-level identity and fine-scale population genetic structure of *C. punctiferalis*, offering a genetic baseline for future regional population comparisons and population genetic studies of this species.

## Materials and Methods

### Insect Sampling and DNA Extraction

Adult males were collected from 13 geographic locations, comprising 12 locations in Korea and 1 location in Japan, from late July to late September 2022 using sex-pheromone-baited traps (Table 1). We aimed to include approximately 30 individuals from each location in the microsatellite analysis and used all available individuals when fewer than 30 were collected. Korean populations were sampled using delta-type sticky traps baited with the 4-component pheromone blend described by Nam et al. (2022), consisting of 0.9 mg *E*10-16: Ald, 0.1 mg *Z*10-16: Ald, 1 mg *Z*3, *Z*6, *Z*9-23: HC, and 1 mg *Z*9-27: HC per rubber septum. The Japanese population was sampled using delta-type sticky traps baited with a commercial *C. punctiferalis* monitoring lure containing *E*10-16: Ald and *Z*10-16: Ald (Sankei Chemical Co., Ltd., Kagoshima, Japan). All collected specimens were identified as *C. punctiferalis* based on adult external morphological characters following Inoue and Yamanaka (2006). Japanese specimens were included to provide comparative material for COI-based phylogenetic and molecular operational taxonomic unit (MOTU)-delimitation analyses and to assess whether the newly developed microsatellite markers could be consistently applied beyond Korean populations. In the microsatellite analyses, the Japanese population was included as a regional reference together with the 12 Korean populations. Genomic DNA (gDNA) was extracted from the leg tissue of individual moths using the Qiagen Gentra Puregene Tissue Kit (Qiagen, Gaithersburg, Maryland, United States) according to the manufacturer’s protocol. The quality and concentration of extracted gDNA were measured using an ND-1000 spectrophotometer (Nanodrop Technologies, Wilmington, Delaware, United States), and samples were stored at −20 °C until further analysis.

**Table 1.** Sampling information of *Conogethes punctiferalis* populations

Sampling location	Code	Country	Latitude (N)	Longitude (E)	Sample size (*n*)
**Paju**	PJ	Korea	37.8505	126.9012	30
**Suwon**	SW	Korea	37.2452	126.9250	29
**Chungju**	CJ	Korea	37.0315	127.8690	30
**Sejong**	SJ	Korea	36.5205	127.2345	20
**Gongju**	GJ	Korea	36.5630	126.9482	24
**Daegu**	DG	Korea	35.8820	128.6255	13
**Cheongdo**	CD	Korea	35.6315	128.6105	10
**Hamyang**	HY	Korea	35.5902	127.7123	30
**Sancheong**	SC	Korea	35.3402	128.0449	31
**Gurye**	GR	Korea	35.2150	127.5350	19
**Gwangyang**	GY	Korea	35.0921	127.7605	30
**Suncheon**	SuC	Korea	35.0305	127.3512	30
**Kasama**	JP	Japan	36.3542	140.2875	30

### COI Amplification, Sequencing, and Dataset Preparation

The mitochondrial COI gene was amplified using the universal primer pair LCO1490 (5′-GGTCAACAAATCATAAAGATATTGG-3′) and HCO2198 (5′-TAAACTTCAGGGTGACCAAAAAATCA-3′) (Folmer et al. 1994). PCR amplification was performed using the AccuPower PCR Premix Kit (Bioneer, Daejeon, Korea) in a total volume of 20 µL containing 1 µL of genomic DNA, 0.75 µL of each primer, and 17.5 µL of distilled water. PCR amplification was performed under the following thermal cycling conditions: an initial denaturation at 95 °C for 3 min, followed by 35 cycles of denaturation at 95 °C for 30 s, annealing at 53 °C for 30 s, and extension at 72 °C for 1 min, with a final extension at 72 °C for 5 min. PCR products were sequenced with an ABI Prism 3730 DNA Sequence Analyzer (Applied Biosystems, Foster City, California, United States) at the National Instrumentation Center for Environmental Management (NICEM), Seoul, Korea. In total, 68 Korean COI sequences were included in the analyses, with at least 5 individuals selected from each locality represented in the COI dataset. The 68 Korean and 6 Japanese COI sequences generated in this study were deposited in GenBank under accession numbers PZ766022 to PZ766095 (Supplementary Table S1). These newly generated sequences were included in the country-level comparisons.

For comparative analyses, publicly available COI sequences of *C. punctiferalis* and other *Conogethes* species were obtained from NCBI GenBank and combined with the sequences generated in this study (Supplementary Table S1). The *C. punctiferalis* dataset comprised 248 sequences from 3 countries, including 68 sequences from Korea, 115 from China, and 65 from Japan. The Chinese sequences represented populations collected from multiple host plants, including chestnut, maize, sorghum, and sunflower (Dong et al. 2023), whereas the Japanese sequences included 6 sequences generated in this study and publicly available sequences collected from various host plants (Aoshima et al. 2020). Additional COI sequences of congeners were included for interspecific comparison within *Conogethes*. Two sequences of *Palpita hypohomalia* (MN609055 and MN609087) were used as outgroups.

To retain the geographically broad Chinese and Japanese *C. punctiferalis* sequences together with reference sequences from 8 other *Conogethes* species, we trimmed all sequences to their shared 254 bp COI region. The 115 Chinese reference sequences (JQ363751.1 to JQ363866.1, excluding JQ363818.1) began farther downstream, whereas 7 congeneric reference sequences (JX064422.1 to JX064428.1) ended earlier, defining the common overlap. This region was used for ML phylogenetic reconstruction and ASAP delimitation, and the corresponding region of *C. punctiferalis* was used for haplotype analysis so that lineage placement and geographic variation could be interpreted together.

### COI-Based Molecular Analyses

COI sequences were aligned and trimmed using ClustalW implemented in BioEdit 7.2 (Thompson et al. 1994, Hall 1999). Maximum-likelihood phylogenetic analysis was conducted using IQ-TREE v. 3.1.3 (Wong et al. 2026). The best-fit nucleotide substitution model was selected according to the Bayesian information criterion using ModelFinder (Kalyaanamoorthy et al. 2017), which identified TPM2u + F + I + R2 as the optimal model. Branch support was assessed using 1,000 ultrafast bootstrap replicates (Hoang et al. 2018).

To evaluate whether Korean populations were assignable to *C. punctiferalis* sensu stricto and whether geographically distinct populations from Korea, China, and Japan were delimited as the same species-level molecular unit or as independent COI-based lineages, MOTUs were inferred using assemble species by automatic partitioning (ASAP), a pairwise distance-based approach. ASAP analysis was performed using ASAPy version 1.0 based on K2P genetic distances (Puillandre et al. 2021).

The numbers of variable and parsimony-informative sites, haplotype diversity, nucleotide diversity (π), and the average number of pairwise nucleotide differences were calculated for the 248 *C. punctiferalis* sequences using DnaSP version 6 (Rozas et al. 2017). A haplotype network was reconstructed using the TCS network method implemented in PopART version 1.7 (Leigh and Bryant 2015).

### Development of Microsatellite Markers and Genotyping

To develop microsatellite markers for *C. punctiferalis*, publicly available larval transcriptome data (SRA accession SRR12988915: Jing et al. 2022) were obtained from the NCBI Sequence Read Archive and processed using the Galaxy platform. Raw reads were quality-trimmed using Trimmomatic with the following settings: SLIDINGWINDOW: 4:20, MINLEN: 50, LEADING: 3, TRAILING: 3, and AVGQUAL: 25 (Bolger et al. 2014). The trimmed reads were subsequently assembled de novo using Trinity with default settings (Grabherr et al. 2011). Microsatellite motifs were identified using PolyMorphPredict, which implements the MISA algorithm (Thiel et al. 2003, Das et al. 2019). The minimum repeat thresholds were set to 6 repeat units for dinucleotide motifs and 5 repeat units for tri-, tetra-, penta-, and hexanucleotide motifs. From the assembled transcripts containing SSRs, 40 SSR-containing loci were selected as primer candidates. Primers were manually designed to be 20 to 24 bp in length, with GC contents of approximately 35% to 60% and melting temperatures of 57.6 to 62.5 °C. Expected amplicon sizes ranged from 97 to 365 bp (Supplementary Table S2).

The candidate loci were first screened individually by singleplex PCR for identifiable amplification products near their expected sizes. PCR amplification was performed in a total volume of 20 μL using AccuPower PCR Premix (Bioneer, Daejeon, Republic of Korea), with each reaction containing 0.75 μL of forward primer (5 pmol/μL), 0.75 μL of reverse primer (5 pmol/μL), 1 μL of template DNA, and 17.5 μL of sterile distilled water. The thermal cycling conditions consisted of an initial denaturation at 95 °C for 3 min, followed by 38 cycles of denaturation at 95 °C for 30 s, annealing at 56 °C for 30 s, and extension at 72 °C for 1 min, with a final extension at 72 °C for 5 min. Amplified products were visualized on 2% agarose gels, purified, and subjected to Sanger sequencing at the NICEM (Seoul, Republic of Korea) to confirm the target repeat motifs. Candidate loci were evaluated for an identifiable amplification product near the expected size, sequence confirmation of the target SSR motif, fragment profiles from which allele sizes could be assigned unambiguously, and variation in allele sizes among the specimens examined.

The 10 retained microsatellite loci were then combined into 3 multiplex PCR panels. Multiplex panel 1 comprised Cp1, Cp2, Cp3, and Cp4; panel 2 comprised Cp5, Cp6, and Cp7; and panel 3 comprised Cp8, Cp9, and Cp10. Panel composition was based on fluorescent dye and expected product size so that loci within each panel could be distinguished during fragment analysis. For each locus, the forward primer was fluorescently labeled with 6-FAM, HEX, or NED, and the dye assignment for each marker is provided in Table 2. Multiplex PCR was performed using the Takara Taq PCR Kit (Takara Bio Inc., Kyoto, Japan) in a total reaction volume of 10 μL. Each reaction contained 1.0 μL of 10× PCR buffer, 1.0 μL of 2.5 mM dNTP mixture, 0.1 μL of Taq polymerase, 0.2 μL of each forward and reverse primer (10 pmol/μL), 2.0 μL of template DNA, and distilled water to a final volume of 10 μL. The thermal cycling conditions consisted of an initial denaturation at 95 °C for 3 min, followed by 39 cycles of denaturation at 95 °C for 30 s, annealing at 56 °C for 30 s, and extension at 72 °C for 30 s, with a final extension at 72 °C for 5 min. For fragment analysis, 1 μL of each PCR product was mixed with 8.5 μL of Hi-Di formamide and 0.5 μL of GeneScan ROX 500 size standard (Applied Biosystems). Fragment separation was performed on an ABI 3730xl Genetic Analyzer, and allele sizes were scored using GeneMapper v. 3.7 (Applied Biosystems, Waltham, Massachusetts, United States).

**Table 2.** Characteristics of 10 novel microsatellite loci developed for *C. punctiferalis*

Locus	Primer sequence (5′–3′)	Repeat motif	*T*_a_ ^a^ (°C)	Expected product size (bp)	*N*_A_ ^b^	*H*_E_ ^c^	*H*_O_ ^d^	*I* ^e^
**Cp1**	F: GACGTATGATTGCACCGACC + 6-FAM R: CCTGGCGTCACTTCACAACT	(AT)_6_	56	297	6.46	0.70	0.86	1.49
**Cp2**	F: ACCATCGGAAGCACTGTTATG+HEX R: TGTAGCTCTATCGCACGCTC	(CA)_8_	56	251	6.31	0.69	0.73	1.46
**Cp3**	F: AGCAAGGAAGACAGCGCAAG+NED R: CGCTCCCGTCATCATCACTA	(TGA)_6_	56	242	7.31	0.75	0.81	1.62
**Cp4**	F: ATGGACTGACGACCTGGTAG + 6-FAM R: TGTTCCCGACCACGTTGACT	(TGA)_7_	56	187	7.23	0.72	0.75	1.54
**Cp5**	F: CAATGTAATCGCTAATCCGTTAG+NED R: TTCGCGCAACGCATCGTAAC	(TA)_10_	56	224	6.77	0.64	0.69	1.34
**Cp6**	F: CTCCAAATGGGCAAACGGAG+HEX R: ATGGAGGCTTCCTCGCTACA	(AT)_8_	56	295	7.92	0.73	0.79	1.54
**Cp7**	F: ACCAACTGAGCCCTTACAGC + 6-FAM R: AGACTCGTCACTATGACCGC	(GAA)_5_	56	238	6.00	0.58	0.64	1.15
**Cp8**	F: TGCGCAAGAAGGAGAAGGAG+NED R: CCCTAGTTCGTCGGGTGTAT	(GAT)_6_	56	255	6.77	0.73	0.78	1.54
**Cp9**	F: GAACCCTCGAGCCTTGTTTC+HEX R: TGTGCCGACCCCACTTAGAG	(ATC)_5_	56	127	5.46	0.66	0.70	1.31
**Cp10**	F: ATCCGCCTGCTTCTTGAGTG + 6-FAM R: CCTACGAGCATCTGACCAGA	(TA)_9_	56	265	7.92	0.75	0.78	1.68

a *T*_a_, annealing temperature.

b *N*_A_, mean number of alleles observed across 13 populations.

c *H*_E_, mean expected heterozygosity.

d *H*_O_, mean observed heterozygosity.

e *I*, mean Shannon’s information index.

### Population Genetic Analysis

Genetic diversity indices, including the number of alleles (*N*_A_), observed (*H*_O_) and expected (*H*_E_) heterozygosities, Shannon’s information index (*I*), and fixation indices (*F*_IS_), were calculated using GenAlEx v. 6.5 (Peakall and Smouse 2012). To estimate uncertainty in population-level *F*_IS_, the 10 locus-specific fixation indices for each population were resampled with replacement 200,000 times. The mean was calculated for each replicate, and the 2.5th and 97.5th percentiles of the resulting values were used as the 95% confidence interval. Potential genotyping errors were assessed using Micro-Checker v. 2.2.3 (Van Oosterhout et al. 2004), and deviations from Hardy–Weinberg equilibrium and genotypic linkage disequilibrium were tested using GENEPOP v. 4.8.6 (Rousset 2008). Exact-test *P*-values were adjusted separately for the 130 Hardy–Weinberg equilibrium tests and the 585 locus-pair–population linkage-disequilibrium tests using the Benjamini–Hochberg false-discovery-rate procedure. Linkage disequilibrium between all pairs of microsatellite loci was evaluated separately for each population using exact tests of genotypic disequilibrium implemented in GENEPOP. Markov chain parameters were set to 10,000 dememorization steps, 100 batches, and 5,000 iterations per batch. The possible effect of null alleles on population differentiation estimates was further evaluated using FreeNA by comparing *F*_ST_ values calculated with and without ENA correction (Chapuis and Estoup 2007). Overall and pairwise *F*_ST_ values were estimated by AMOVA in GenAlEx v. 6.5 using the codominant-allelic distance option with within-individual analysis suppressed. Significance was assessed with 999 permutations for the overall analysis and for each pairwise population comparison. *P*-values from the 78 pairwise comparisons were adjusted using the Benjamini–Hochberg false-discovery-rate procedure. Pairwise Nei’s genetic distances among populations were calculated from SSR allele frequencies across the 10 microsatellite loci using GenAlEx v. 6.5. A UPGMA dendrogram was constructed from the pairwise sample-size-corrected Nei’s standard genetic distances (*D*_ST_) matrix, and node support was assessed using 5,000 bootstrap replicates, with loci resampled with replacement in each replicate using POPTREE2 (Takezaki et al. 2010). Bayesian clustering of microsatellite genotypes was performed using STRUCTURE v. 2.3.2 (Pritchard et al. 2000) under the admixture model with correlated allele frequencies. Values of *K* from 1 to 10 were evaluated using 10 independent runs for each *K*. Each run consisted of a burn-in period of 10,000 iterations followed by 100,000 Markov chain Monte Carlo iterations. The most strongly supported clustering level was evaluated using the Evanno Δ*K* method implemented in STRUCTURE HARVESTER (Evanno et al. 2005, Earl and vonHoldt 2012). Principal coordinate analysis (PCoA) was performed based on codominant genotypic distances to visualize genetic relationships among individuals. Genetic diversity, AMOVA, genetic distance, STRUCTURE, and PCoA analyses were conducted using the full 13-population dataset, with the Japanese population treated as a regional reference. The divMigrate analysis was restricted to the 12 Korean populations. Directional relative migration among the 12 Korean populations was estimated using the divMigrate function implemented in the diveRsity package in R, with Jost’s *D* used as the genetic differentiation statistic (Keenan et al. 2013, Sundqvist et al. 2016). The Japanese population was excluded because this analysis focused specifically on connectivity among Korean populations. Relative directional values were normalized so that the maximum value equaled 1.0. Asymmetry between reciprocal directions was assessed using 1,000 bootstrap replicates and was considered supported when the 95% bootstrap confidence intervals of the reciprocal estimates did not overlap.

## Results

### COI Variation and Lineage Delimitation

Haplotype analysis identified 48 haplotypes within *C. punctiferalis*, with a haplotype diversity of 0.8336. Among the 248 *C. punctiferalis* sequences, 27 variable sites were detected, including 15 parsimony-informative sites. Nucleotide diversity was 0.00827, and the average number of pairwise nucleotide differences was 2.101. Intraspecific pairwise divergence ranged from 0% to 2.76% (mean 0.83%). The haplotype network consisted of several high-frequency haplotypes and multiple low-frequency haplotypes (Fig. 1A). The most frequent haplotype was Hap_3, which included 90 sequences. Hap_20 and Hap_13 were the next most frequent haplotypes, containing 35 and 27 sequences, respectively. Korean COI sequences were distributed among 7 haplotypes, including Hap_3, Hap_13, Hap_20, Hap_41, Hap_42, Hap_47, and Hap_48. Among these haplotypes, Hap_3, Hap_13, Hap_20, Hap_41, and Hap_42 also contained non-Korean reference sequences. Hap_47 and Hap_48 were each represented by 2 Korean sequences. In the haplotype network, Korean sequences were mainly positioned within or near haplotypes that also contained Chinese or Japanese reference sequences.

**Fig. 1.**
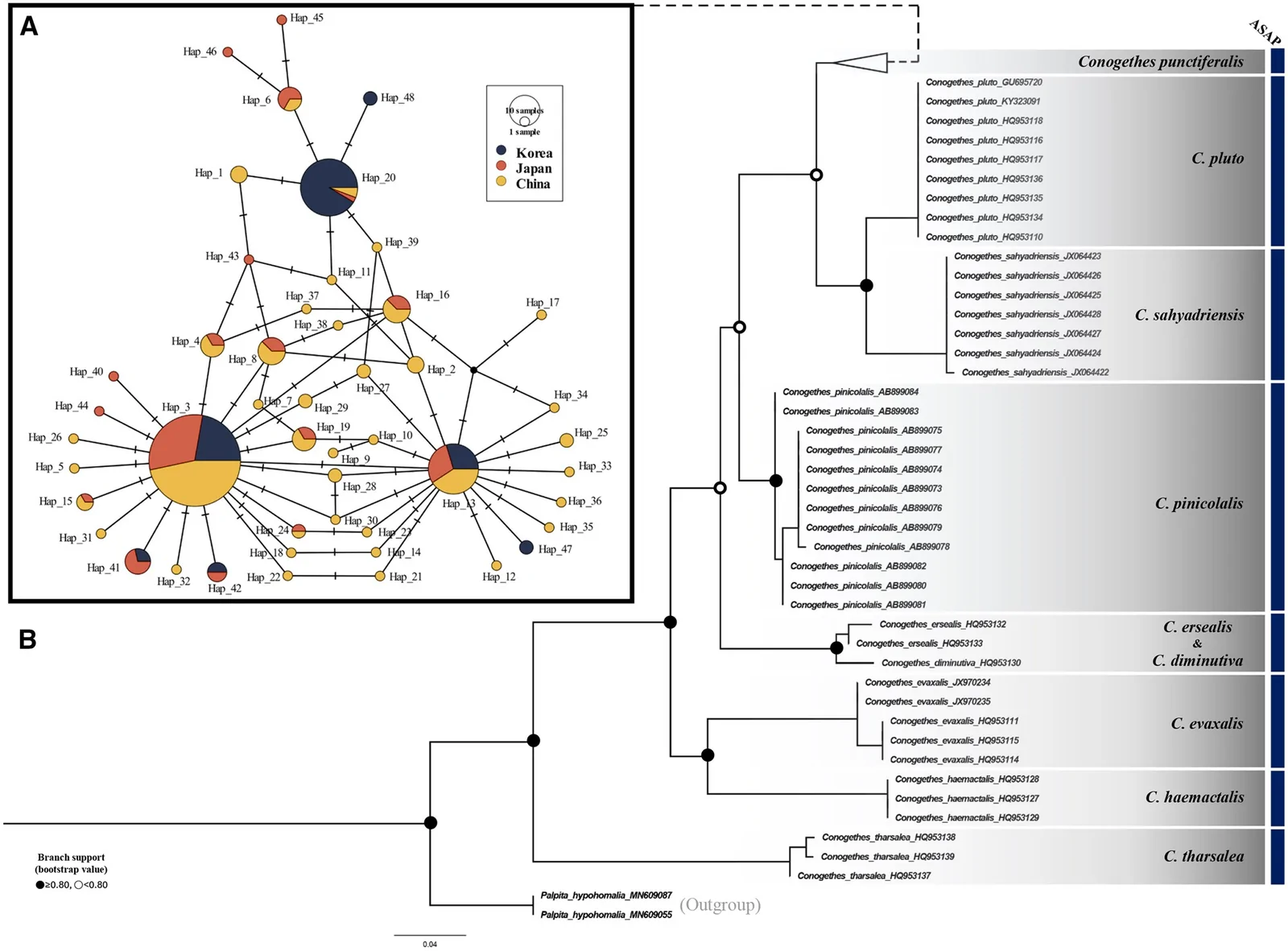
COI haplotype network, maximum-likelihood phylogeny, and ASAP delimitation of *C. punctiferalis* and related taxa. A) TCS haplotype network; circle size indicates haplotype frequency and colors indicate country. B) COI-based ML tree. Filled and open circles indicate bootstrap support ≥80% and <80%, respectively. Blue bars indicate ASAP MOTUs.

The ML phylogeny recovered *C. punctiferalis* as a monophyletic lineage distinct from the other *Conogethes* species included in the analysis (Fig. 1B). Korean samples were nested within this lineage together with Chinese and Japanese samples collected from chestnut and other host plants.

COI-based MOTU delimitation using ASAP divided the COI dataset into 8 MOTUs, with an ASAP score of 1.50 and a distance threshold of 0.0343. All *C. punctiferalis* sequences from Korea, China, and Japan were assigned to a single MOTU. The other *Conogethes* species were assigned to separate MOTUs, except that *Conogethes ersealis* and *Conogethes diminutiva* were grouped together in the same MOTU (Fig. 1B).

### Microsatellite Marker Development and Genotyping Results

De novo assembly of the *C. punctiferalis* larval transcriptome yielded 97,867 assembled transcripts with a total assembled length of 120,885,264 bp. MISA-based screening implemented in PolyMorphPredict identified 8,189 SSRs distributed across 6,713 SSR-containing transcripts, including 1,119 transcripts containing more than 1 SSR and 584 SSRs present in compound formation.

Among the 40 candidate primer pairs designed from SSR-containing transcripts, 10 loci met the screening criteria described above and were retained as microsatellite markers for *C. punctiferalis* (Supplementary Table S2). These loci, designated Cp1 to Cp10, included di- and trinucleotide repeat motifs, with expected fragment sizes ranging from 127 to 297 bp. Using these 10 markers, a total of 326 adult males from 13 geographic populations in Korea and Japan were successfully genotyped for subsequent population genetic analyses.

### Microsatellite Polymorphism and Genetic Diversity

The 10 microsatellite loci exhibited moderate to high levels of polymorphism across the examined populations. The mean number of alleles per locus ranged from 5.46 in Cp9 to 7.92 in Cp6 and Cp10, with an overall mean of 6.82 alleles per locus (Table 2). Mean expected heterozygosity (*H*_E_) ranged from 0.58 in Cp7 to 0.75 in Cp3 and Cp10, whereas mean observed heterozygosity (*H*_O_) ranged from 0.64 in Cp7 to 0.86 in Cp1.

At the population level, the mean number of alleles (*N*_A_) ranged from 4.5 in PJ to 8.8 in SC (Table 3), although these values were interpreted descriptively because sample sizes differed among populations. Expected heterozygosity (*H*_E_) ranged from 0.55 in PJ to 0.81 in SJ, GJ, and SC, whereas observed heterozygosity (*H*_O_) ranged from 0.50 in GY to 0.96 in GJ. Population-level *F*_IS_ estimates ranged from −0.205 in SW to 0.186 in HY (Table 3). The 95% confidence intervals excluded zero for the positive estimates in CJ and GY and the negative estimates in SW, SJ, GJ, DG, CD, SC, and GR. The intervals for PJ, HY, JP, and SuC included zero.

**Table 3.** Genetic diversity indices of 13 *Conogethes punctiferalis* populations based on 10 microsatellite loci

Population	*N* ^a^	*N*_A_ ^b^	*H*_E_ ^c^	*H*_O_ ^d^	*F*_IS_ ^e^	95% CI for *F*_IS_
**PJ**	30	4.5	0.55	0.63	−0.100	−0.488 to 0.302
**SW**	29	8.2	0.79	0.95	−0.205	−0.237 to −0.170
**CJ**	30	6.4	0.66	0.55	0.172	0.081 to 0.280
**SJ**	20	8.1	0.81	0.94	−0.159	−0.198 to −0.120
**GJ**	24	8.2	0.81	0.96	−0.193	−0.238 to −0.151
**DG**	13	6.6	0.75	0.85	−0.134	−0.227 to −0.038
**CD**	10	6.6	0.75	0.85	−0.135	−0.208 to −0.063
**SC**	31	8.8	0.81	0.95	−0.186	−0.241 to −0.135
**GR**	19	6.8	0.75	0.85	−0.123	−0.197 to −0.031
**GY**	30	5.5	0.60	0.50	0.184	0.047 to 0.335
**HY**	30	6.6	0.60	0.51	0.186	−0.001 to 0.358
**JP**	30	6.4	0.65	0.68	−0.031	−0.134 to 0.078
**SuC**	30	5.9	0.62	0.58	0.054	−0.087 to 0.206

Abbreviations: CD, Cheongdo; CJ, Chungju; DG, Daegu; GJ, Gongju; GR, Gurye; GY, Gwangyang; HY, Hamyang; JP, Kasama; PJ, Paju; SC, Sancheong; SJ, Sejong; SuC, Suncheon; SW, Suwon.

a *N*, sample size.

b *N*_A_, mean number of alleles. *N*_A_ is reported descriptively because sample sizes differed among populations.

c *H*_E_, expected heterozygosity.

d *H*_O_, observed heterozygosity.

e *F*_IS_, population-specific fixation index. Positive values indicate heterozygote deficits, whereas negative values indicate heterozygote excess.

Nominal departures from Hardy–Weinberg equilibrium were detected in 68 of 130 locus–population combinations, of which 57 remained significant after Benjamini–Hochberg false-discovery-rate correction (Supplementary Table S3). Micro-Checker analysis did not reveal consistent evidence of null alleles, large-allele dropout, or scoring errors due to stuttering across the examined microsatellite loci. FreeNA estimated nonzero null allele frequencies in some locus–population combinations, but the effect of these estimates on population differentiation was limited. The global *F*_ST_ value decreased only slightly after ENA correction, from 0.1019 without correction to 0.0951 with correction, with broadly overlapping 95% confidence intervals (Supplementary Table S4). Pairwise genotypic linkage-disequilibrium tests identified nominally significant associations in 100 of 585 locus-pair–population combinations. After Benjamini–Hochberg false-discovery-rate correction across all tests, 35 combinations remained significant (Supplementary Table S5). Significant associations were concentrated in SuC (13 combinations), HY (10), CJ (7), and GY (3), with 1 significant association each in SJ and PJ; no significant associations remained in the other 7 populations. No locus pair showed significant linkage disequilibrium across a majority of populations. The Cp6–Cp10 pair was significant in 4 populations, and Cp1–Cp10 was significant in 3 populations, whereas all other significant locus pairs occurred in no more than 2 populations.

### Population Genetic Differentiation and Genetic Structure

AMOVA partitioned 10% of the total genetic variation among populations and 90% within populations (*F*_ST_ = 0.1013, *P* = 0.001; Table 4). The UPGMA dendrogram based on *D*_ST_ separated the sampled populations into 3 broad groups (Fig. 2A). The first group consisted of GJ and SC, the second group included SW, SJ, and CD, and the third group comprised DG, GR, CJ, GY, HY, JP, PJ, and SuC. The Nei’s genetic-distance matrix broadly reflected this grouping pattern (Fig. 2B). Pairwise *F*_ST_ values ranged from 0.000 to 0.206 (Fig. 2B). After Benjamini–Hochberg correction, 73 of the 78 pairwise comparisons were significant. The 5 nonsignificant comparisons were SW–SJ, SW–CD, GJ–SC, SJ–CD, and DG–GR. The highest *F*_ST_ was observed between GJ and PJ (0.206), followed by SC and PJ (0.205).

**Fig. 2.**
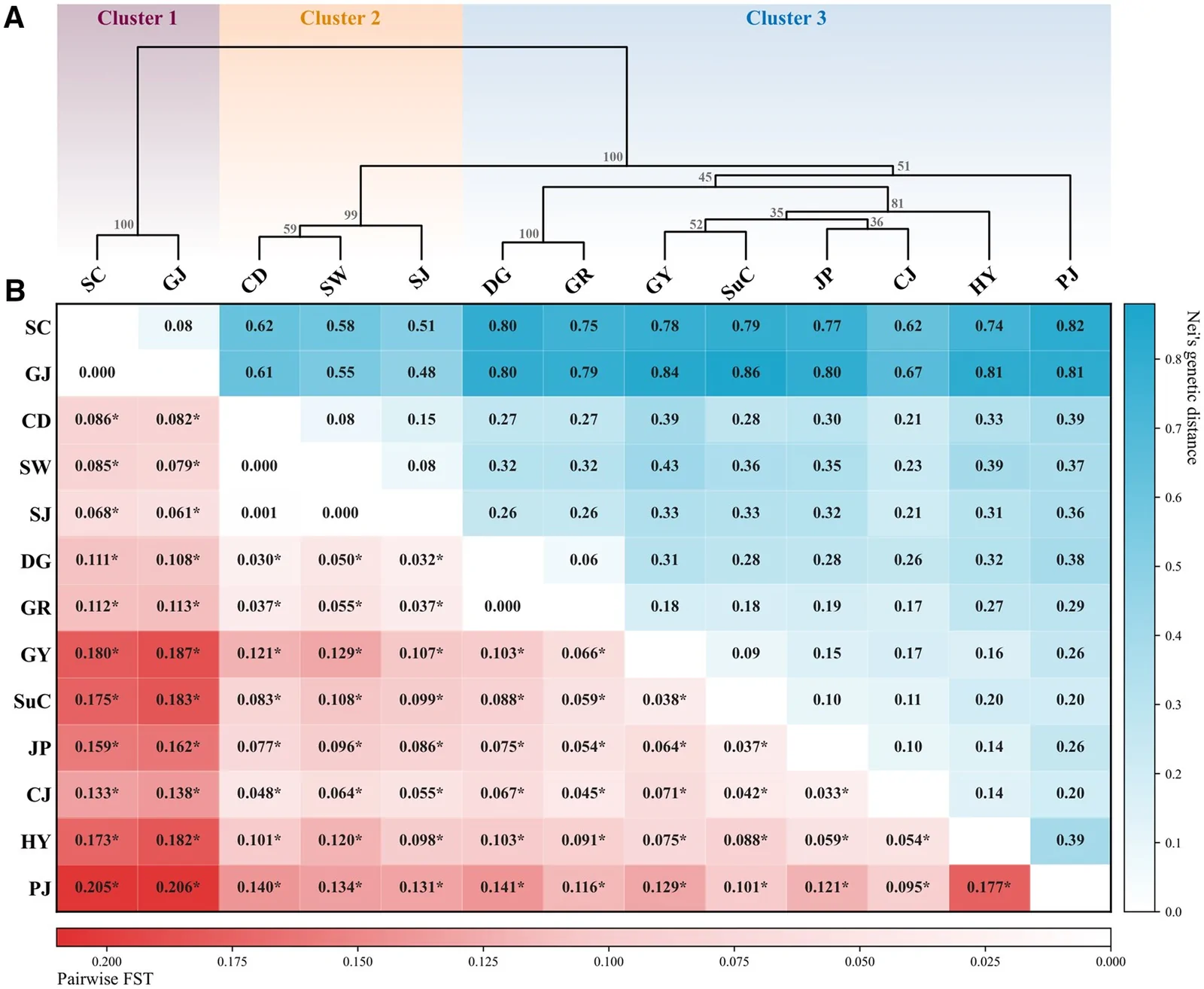
Genetic relationships among *C. punctiferalis* populations based on microsatellite data. A) UPGMA dendrogram based on sample-size-corrected Nei’s standard genetic distances (*D*_ST_) calculated using POPTREE2; node values indicate bootstrap support obtained from 5,000 replicates. B) Pairwise Nei’s genetic distances are shown above the diagonal, and pairwise *F*_ST_ values are shown below the diagonal. An asterisk indicates a pairwise *F*_ST_ comparison significant after Benjamini–Hochberg correction across 78 comparisons (adjusted *P* < 0.05).

**Table 4.** Analysis of molecular variance (AMOVA) for 13 populations of *Conogethes punctiferalis* based on microsatellite loci

Source of variation	Degrees of freedom	Sums of squares	Mean sums of squares	Estimated variance	% of variation	*P*-value
**Among populations**	12	280.196	23.350	0.398	10	0.001
**Within populations**	639	2255.430	3.530	3.530	90	–
**Total**	651	2535.626	–	3.928	100	–

*P*-values were obtained by permutation tests.

The highest Δ*K* value was observed at *K* = 3 based on the Evanno Δ*K* method (Fig. 3A). Across *K* = 2, 3, and 4, however, individual assignment patterns were highly admixed, and no geographic population was composed exclusively of a single inferred genetic cluster. PCoA based on codominant genotypic distances was conducted for 326 individuals from 13 populations (Fig. 3B). The first 2 principal coordinates explained 11.56% and 6.13% of the total variation, respectively, accounting for 17.69% combined. In this two-dimensional projection, most individuals overlapped across populations, whereas individuals from GJ and SC were slightly displaced from the main distribution.

**Fig. 3.**
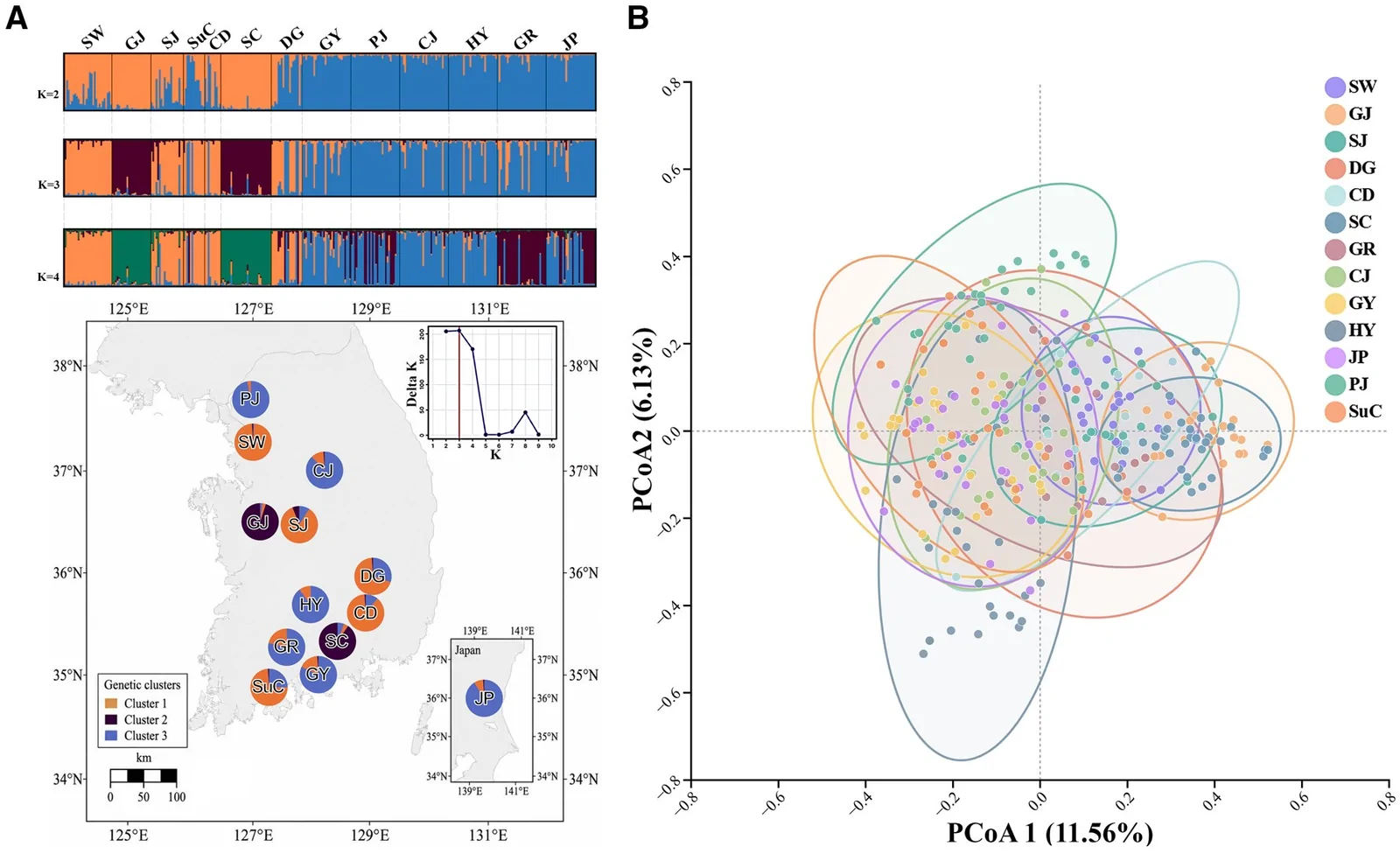
Microsatellite-based population genetic structure of *C. punctiferalis*. A) STRUCTURE results for *K* = 2 to 4 and geographic distribution of mean cluster membership at *K* = 3 across 12 Korean populations and the Japanese reference population. Each vertical bar represents an individual, and colors indicate membership in the inferred genetic clusters. Pie charts show the mean cluster membership proportions of each population. The inset shows Δ*K* across the tested values of *K*, with the highest value observed at *K* = 3. B) PCoA of individual microsatellite genotypes. Points are colored by population, and ellipses depict population-level dispersion. The first 2 axes explain 11.56% and 6.13% of the total variation, respectively.

### Directional Relative Connectivity Among Korean Populations

Directional relative migration was evaluated among the 12 Korean populations, excluding the Japanese reference population. Bootstrap-supported directional asymmetries were detected among several population pairs (Fig. 4; Supplementary Table S6). Among the supported directions, relative migration values ≥0.30 were observed from CJ to SW (0.427) and SC (0.315), and from PJ to SW (0.374), SJ (0.354), DG (0.342), CD (0.366), SC (0.399), and GR (0.576). Of these, the highest supported relative migration value was observed from PJ to GR.

**Fig. 4.**
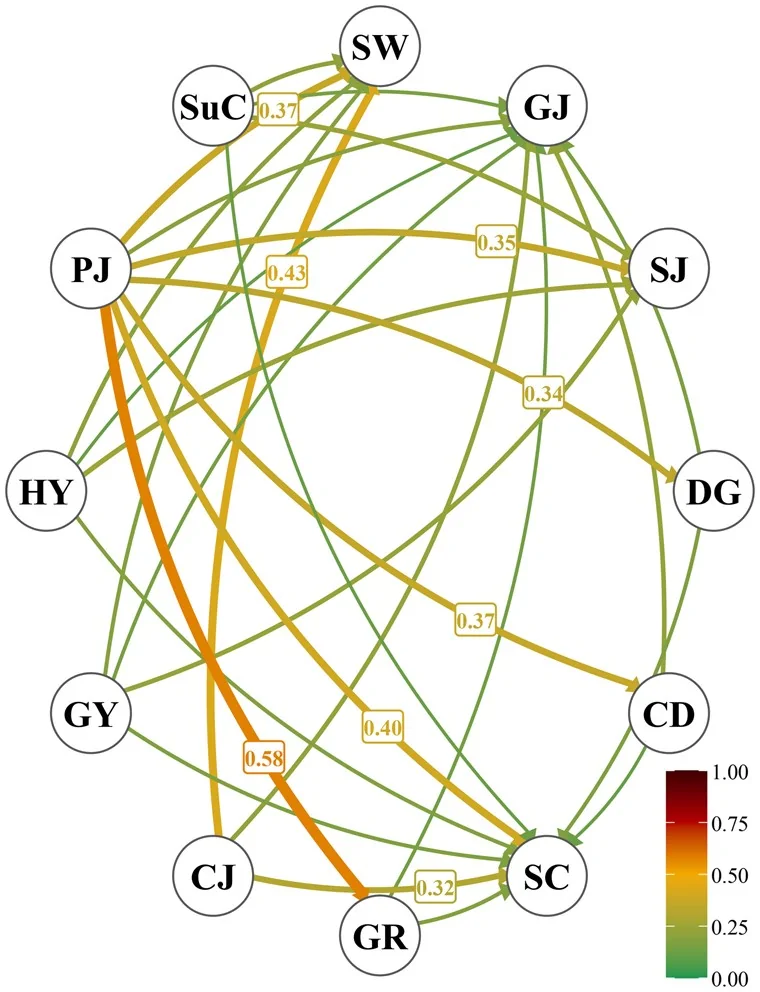
Bootstrap-supported directional relative migration network among 12 Korean populations of *C. punctiferalis*. The network was estimated from microsatellite data using divMigrate with Jost’s *D* and 1,000 bootstrap replicates; the Japanese reference population was excluded. Arrows indicate source-to-recipient directional relative migration, with width and color (green–red) proportional to the relative value. Only directions supported by nonoverlapping 95% bootstrap confidence intervals are shown. Values are labeled when ≥0.30.

## Discussion

### COI-Based Genetic Identity of Korean Populations

The COI-based phylogeny and ASAP analysis broadly recovered distinct genetic lineages and MOTUs among the examined *Conogethes* taxa. *Conogethes punctiferalis* formed a monophyletic lineage, and all individuals from Korea, China, and Japan were assigned to a single COI-based MOTU (Fig. 1B). Most of the other nominal species were assigned to separate MOTUs, whereas *C. ersealis* and *C. diminutiva* were grouped within the same MOTU. This result is consistent with previous reports of their close COI affinity and uncertain taxonomic boundary (Shashank et al. 2018), although their taxonomic status cannot be resolved here because only a few reference sequences and a single mitochondrial marker were analyzed. Within *C. punctiferalis*, Korean individuals were included in major haplotypes shared across East Asia or were positioned close to them in the haplotype network (Fig. 1A). These results indicate that the Korean populations examined in this study do not form an independent mitochondrial lineage distinct from *C. punctiferalis* sensu stricto.

These findings distinguish the Korean populations from the divergent mitochondrial lineages previously reported within nominal *C. punctiferalis*. An et al. (2024) showed that some sequences identified as *C. punctiferalis* formed a *C. cf. ­punctiferalis* lineage exhibiting approximately 5.91% COI sequence divergence from *C. punctiferalis* sensu stricto and suggested that this lineage may represent a separate putative species. In contrast, the Korean individuals examined here clustered with East Asian reference sequences treated as *C. punctiferalis* sensu stricto in the present dataset.

Furthermore, the assignment of Korean, Chinese, and Japanese individuals to a single COI-based MOTU suggests that the reported differences in pheromone blends or lure responses among the 3 countries do not correspond directly to country-specific deep mitochondrial lineages. In this context, Aoshima et al. (2020) identified 2 COI clades in Japan but found no significant difference in clade frequency between specimens captured using different pheromone lures and those collected directly from host plants. They also observed random mating between the 2 clades. Thus, differences in mitochondrial lineage do not necessarily translate into differences in pheromone attractiveness or reproductive isolation. Nevertheless, because the causes of geographic variation in pheromone composition among Korean, Chinese, and Japanese populations were not directly evaluated in this study, further investigation is needed to determine whether such variation is associated with nuclear genetic variation, local adaptation, or environmental factors within *C. punctiferalis* sensu stricto.

### Development and Reliability of Transcriptome-Derived SSR Markers

We developed 10 novel microsatellite markers for *C. punctiferalis* through transcriptome assembly and SSR screening. Of the 40 candidate loci, 10 were selected based on amplification success, clear fragment profiles, and polymorphism, and these loci exhibited moderate to high levels of polymorphism across the examined populations. The mean number of alleles per locus ranged from 5.46 to 7.92, and expected heterozygosity ranged from 0.58 to 0.75, providing sufficient variation for assessing population-level genetic diversity and differentiation (Table 2). Their consistent amplification in both Korean and Japanese individuals also indicates their potential utility for future comparisons among regional populations.

Transcriptome- or EST-derived SSRs are developed from the relatively conserved flanking regions of transcribed sequences and therefore often show high transferability among populations of the same species and, in some cases, among closely related taxa (Ellis and Burke 2007). Although cross-species amplification of SSR markers has been reported in other insects (An et al. 2014, Liu et al. 2022), the transferability of the 10 loci developed here to other *Conogethes* species remains to be tested. In insects, comparisons between EST-derived and genomic SSRs have also shown that EST-SSRs can provide broadly comparable estimates of population genetic diversity, *F*_ST_, and population assignment (Kim et al. 2008). The markers developed in this study therefore expand the available novel nuclear marker resources for *C. punctiferalis* and provide useful tools for comparing genetic diversity and fine-scale population structure among geographic populations. The 3 multiplex panels may also facilitate repeated genotyping of additional geographic or temporal samples using the same 10 loci. For future applications, universal fluorescent labeling with tailed locus-specific primers could reduce primer-labeling costs, although the modified assay would require optimization and validation (Blacket et al. 2012). For repeated population surveys, applying this established assay to new samples may be cost-efficient relative to generating genome-wide SNP data, depending on sample numbers and available genotyping facilities (Puckett 2017, Lemopoulos et al. 2019). In addition, access to NGS resources may vary among research settings (Getchell et al. 2024). Where access is limited, this established SSR panel may offer an alternative for repeated population surveys.

Nevertheless, nominal departures from Hardy–Weinberg equilibrium were detected in 68 of 130 locus–population ­combinations, of which 57 remained significant after false-discovery-rate correction (Supplementary Table S3). The occurrence and direction of these departures varied among loci and populations, with both heterozygote deficits and excesses observed, indicating that no single process adequately explained the overall pattern. Micro-Checker detected no consistent evidence of null alleles, large-allele dropout, or scoring errors caused by stuttering, and no locus showed uniform departure across all populations. Moreover, ENA correction reduced the global *F*_ST_ estimate only slightly, from 0.1019 to 0.0951, with broadly overlapping 95% confidence intervals (Supplementary Table S4). Pairwise linkage-disequilibrium tests likewise showed no pervasive association among loci: after false-discovery-rate correction, 35 of 585 locus-pair–population tests remained significant, and these associations were largely restricted to particular populations rather than consistently repeated throughout the dataset (Supplementary Table S5). Taken together, these results suggest that population- and sample-specific factors may have contributed to some departures from equilibrium, while providing limited evidence for systematic genotyping artifacts or widespread nonindependence across the marker panel.

Overall, these results support the applicability of the 10 loci as an informative multilocus marker set for assessing genetic diversity, population differentiation, and regional genetic structure in *C. punctiferalis*.

### Population Genetic Structure and Connectivity

Microsatellite analyses of the full 13-population dataset, including 1 Japanese regional reference population, indicated weak but detectable population-level structuring rather than complete panmixia. Within this broader framework, the Korean populations showed extensive admixture and only weak regional tendencies. Pairwise *F*_ST_ values ranged from 0.000 to 0.206, indicating generally low to moderate differentiation (Fig. 2B). STRUCTURE analysis showed the highest Δ*K* value at *K* = 3, although individual assignment patterns remained highly admixed and the proportional composition of inferred genetic components differed among populations. At *K* = 3, GJ and SC showed similar component compositions, as did SW, SJ, and CD (Fig. 3A). These similarities indicate regional tendencies in genetic composition rather than discrete genetic boundaries among populations. Consistent with this pattern, the UPGMA dendrogram based on *D*_ST_ separated the sampled populations into 3 broad groups comprising GJ–SC, SW–SJ–CD, and the remaining populations (Fig. 2A).

At the individual level, however, most populations comprised mixtures of multiple inferred genetic components rather than a single component. Thus, although the proportional genetic composition differed among populations, the regional populations were not clearly separated genetically. The observed pattern is therefore better interpreted as weak population-level structuring in the presence of extensive individual-level admixture rather than as 3 clearly isolated regional populations. In the PCoA, individuals from GJ and SC tended to be slightly displaced from the main distribution, whereas most individuals overlapped across populations on the first 2 coordinates (Fig. 3B). Because these coordinates explained only 17.69% of the total variation, the apparent overlap and separation in this projection should be interpreted cautiously. The STRUCTURE results provide the primary evidence for individual-level admixture. AMOVA also showed that 90% of the total genetic variation occurred within populations, whereas only 10% was attributable to variation among populations (Table 4). Thus, although allele-frequency differences were present among the sampled populations, these differences did not correspond to strong population isolation. This interpretation is broadly consistent with previous studies reporting weak geographic structuring in *C. punctiferalis*. Studies using ISSRs, mitochondrial and nuclear sequences, and genome-wide SNPs have generally detected limited differentiation among geographic populations (Zhang et al. 2010, Aoshima et al. 2020, Dong et al. 2023, Gao et al. 2023). In particular, an ISSR-based analysis of 11 Chinese populations revealed low genetic differentiation and high gene flow, closely resembling the combination of weak regional structuring and extensive connectivity observed in the present study (Zhang et al. 2010). The similarly limited overall differentiation reported from genome-wide SNP data in China provides a further point of comparison for the Korean population patterns identified with our SSR panel (Gao et al. 2023). Collectively, these findings indicate that *C. punctiferalis* generally maintains high regional genetic connectivity despite weak local genetic differences. Nevertheless, high overall connectivity does not necessarily preclude local genetic patterning. Dong et al. (2023) found no clear host-associated differentiation between maize and orchard populations but reported that landscape composition, including surrounding cropland and noncrop areas, was associated with the frequencies of some mitochondrial haplotypes. This finding suggests that landscape heterogeneity may influence local genetic composition even when overall gene flow remains high. Accordingly, the weak population-level structuring detected in the present study may reflect local differences in host availability, landscape configuration, or demographic history. However, these ecological and landscape factors were not directly evaluated in this study and require further investigation. The individual-level admixture was broadly consistent with the trans-regional relative-connectivity patterns indicated by divMigrate. Bootstrap-supported directional asymmetries were detected among several Korean population pairs (Fig. 4; Supplementary Table S6), indicating that relative connectivity was directionally heterogeneous among some population pairs. Because divMigrate derives standardized directional relative migration from genetic differentiation, these patterns should be interpreted as comparative indicators of connectivity rather than as direct estimates of contemporary migration rates.

The Japanese population was included only as a regional reference and did not form a discrete group in the UPGMA, STRUCTURE, or PCoA analyses. Its inclusion provided cross-regional context for the Korean data and demonstrated that the markers could be applied beyond Korean populations. However, because only 1 Japanese locality was sampled, no inference can be made regarding population structure within Japan. Moreover, different pheromone blends were used for sampling in Korea and Japan. Pheromone composition and individual responses to these blends were not measured, so our genetic data cannot establish whether the reported behavioral differences are associated with genetic variation. Therefore, the Japanese sample should not be interpreted as a standardized between-country comparison of lure-responsive phenotypes. Overall, Korean *C. punctiferalis* populations appear to represent neither a panmictic population nor 3 clearly separated regional populations. The UPGMA and STRUCTURE analyses indicated weak but detectable regional tendencies, whereas individual-level admixture in STRUCTURE, broad overlap in the PCoA, and high within-population variation in the AMOVA were consistent with extensive connectivity among Korean populations. The divMigrate analysis additionally identified bootstrap-supported directional asymmetries among some population pairs, indicating that relative connectivity was not uniform in direction. Korean populations are therefore best characterized as exhibiting weak regional structure under extensive admixture and connectivity.

Taken together, these findings suggest that Korean populations are characterized by extensive genetic connectivity with weak but detectable regional differentiation. Accordingly, regional populations should not automatically be treated as fully independent management units, although localized genetic differences may still occur within this broadly connected system. The microsatellite markers developed here provide a baseline for future temporal and spatial sampling to evaluate the stability of these patterns, recolonization processes, and the geographic distribution of independently characterized management-relevant traits. Such longitudinal sampling will be necessary to determine whether the weak regional genetic patterns detected in this study persist over time.

In this study, COI-based phylogenetic and MOTU-delimitation analyses placed Korean *C. punctiferalis* populations within *C. punctiferalis* sensu stricto together with populations from China and Japan, indicating that they do not represent a distinct country-specific mitochondrial lineage. We also developed 10 polymorphic transcriptome-derived microsatellite markers and applied them to population-level analyses. Across the full 13-population dataset, including 1 Japanese regional reference population, microsatellite data revealed weak regional structuring and extensive individual-level admixture, whereas the Korea-only divMigrate analysis indicated heterogeneous trans-regional relative connectivity. Importantly, the newly developed SSR markers expand the limited nuclear marker resources available for *C. punctiferalis* and provide a practical tool for future assessments of genetic diversity, fine-scale population structure, connectivity, and population monitoring across geographic regions.

## Supplementary Material

ieag124_Supplementary_Data

## Data Availability

Raw datasets generated during the current study are available from the corresponding author on reasonable request.
