# Supplementary material for: Genetic characterization and development of novel microsatellite markers for Conogethes punctiferalis (Lepidoptera: Crambidae) populations in the Republic of Korea
Source: J Insect Sci. 2026 Nov 14;26(6):ieag124. doi: 10.1093/jisesa/ieag124 (PMC13769238; doi:10.1093/jisesa/ieag124)
Supplement: Supplementary file 1 [file ieag124_supplementary_data.docx]

**Supplementary Table S1.** List of COI sequences and corresponding GenBank accession numbers used in the molecular analyses of *Conogethes punctiferalis* and related taxa.

| **Taxon** | **Country/source group** | **Source** | **Gene** | **n** | **GenBank accession number(s)** |
| --- | --- | --- | --- | --- | --- |
| *Conogethes punctiferalis* | Korea (this study) | This study | COI | 68 | PZ766022–PZ766048; PZ766055–PZ766095 |
| *Conogethes punctiferalis* | Japan (this study) | This study | COI | 6 | PZ766049–PZ766054 |
| *Conogethes punctiferalis* | Japan (GenBank) | GenBank | COI | 59 | AB899096.1-AB899154.1 |
| *Conogethes punctiferalis* | China (GenBank) | GenBank | COI | 115 | JQ363751.1-JQ363817.1; JQ363819.1-JQ363866.1 |
| *Conogethes diminutiva* | Reference taxon (GenBank) | GenBank | COI | 1 | HQ953130.1 |
| *Conogethes ersealis* | Reference taxon (GenBank) | GenBank | COI | 2 | HQ953132.1-HQ953133.1 |
| *Conogethes evaxalis* | Reference taxon (GenBank) | GenBank | COI | 5 | HQ953111.1; HQ953114.1-HQ953115.1; JX970234.1-JX970235.1 |
| *Conogethes haemactalis* | Reference taxon (GenBank) | GenBank | COI | 3 | HQ953127.1-HQ953129.1 |
| *Conogethes pinicolalis* | Reference taxon (GenBank) | GenBank | COI | 12 | AB899073.1-AB899084.1 |
| *Conogethes pluto* | Reference taxon (GenBank) | GenBank | COI | 9 | GU695720.1; HQ953110.1; HQ953116.1-HQ953118.1; HQ953134.1-HQ953136.1; KY323091.1 |
| *Conogethes sahyadriensis* | Reference taxon (GenBank) | GenBank | COI | 7 | JX064422.1-JX064428.1 |
| *Conogethes tharsalea* | Reference taxon (GenBank) | GenBank | COI | 3 | HQ953137.1-HQ953139.1 |
| *Palpita hypohomalia* | Outgroup (GenBank) | GenBank | COI | 2 | MN609055.1; MN609087.1 |

**Supplementary Table S2.** Information on 40 candidate primer sets for *C. punctiferalis*.

| **No.** | **Sequence name** | **Repeat** | **Name** | **Primer sequence (5′–3′)** | **Tm (ºC)** | **Expected product size (bp)** |
| --- | --- | --- | --- | --- | --- | --- |
| 1 | TRINITY_DN1118_c0_g1_i9 | (TA)_12_ | CpTest1_F | TAAGGGAGCGACCTGTCATC | 60.5 | 254 |
|  |  |  | CpTest1_R | GTGGACGTAAATATATGTCGGAG | 60.9 |  |
| 2 | TRINITY_DN6121_c0_g1_i1 | (CA)_13_ | CpTest2_F | GTTAGCAACCGACTGGTTAAAG | 60.1 | 245 |
|  |  |  | CpTest2_R | CCGGAAATAGGTACAGGTAGG | 61.2 |  |
| 3 | TRINITY_DN23_c1_g1_i2 | (CGGA)_13_ | CpTest3_F | GGGAGTTCGTCGAGTAATCG | 60.5 | 243 |
|  |  |  | CpTest3_R | TCACGCGATGTGATCGACAC | 60.5 |  |
| 4 | TRINITY_DN2111_c0_g1_i2 | (TAAA)_9_ | CpTest4_F | TACGGATGGCTATGTTATCGC | 59.5 | 127 |
|  |  |  | CpTest4_R | CGATGCGAGAATGTATGCGC | 60.5 |  |
| 5 | TRINITY_DN14846_c0_g1_i2 | (CA)_14_ | CpTest5_F | ACACCCATAGTCGCACACAG | 60.5 | 314 |
|  |  |  | CpTest5_R | GTACACTTCGCGTGAGGTAC | 60.5 |  |
| 6 | TRINITY_DN23_c1_g1_i2 | (CGGA)_13_ | CpTest6_F | GGGAGTTCGTCGAGTAATCG | 60.5 | 196 |
|  |  |  | CpTest6_R | CGTAAGTAAGGTGTGCGTCG | 60.5 |  |
| 7 | TRINITY_DN3301_c0_g1_i2 | (TAAA)_7_ | CpTest7_F | ACATTGAATGCCGTAACAGCC | 59.5 | 278 |
|  |  |  | CpTest7_R | CTAAGAACACGCCAGGTGTG | 60.5 |  |
| 8 | TRINITY_DN948_c0_g1_i6 | (TTCTA)_6_ | CpTest8_F | ATCTGATACTGATGCTGTCGC | 59.5 | 171 |
|  |  |  | CpTest8_R | TATCTGACAGCGACTGTACAG | 59.5 |  |
| 9 | TRINITY_DN9022_c0_g1_i3 | (GT)_24_ | CpTest9_F | ACTGTGCACGCAAAGAGTGG | 60.5 | 257 |
|  |  |  | CpTest9_R | CAAGGTAACGCCCGACACAT | 60.5 |  |
| 10 | TRINITY_DN798_c1_g1_i1 | (AT)_6_ | Cp1_F | GACGTATGATTGCACCGACC | 60.5 | 297 |
|  |  |  | Cp1_R | CCTGGCGTCACTTCACAACT | 60.5 |  |
| 11 | TRINITY_DN6695_c0_g1_i2 | (CA)_8_ | Cp2_F | ACCATCGGAAGCACTGTTATG | 59.5 | 251 |
|  |  |  | Cp2_R | TGTAGCTCTATCGCACGCTC | 60.5 |  |
| 12 | TRINITY_DN6518_c0_g1_i2 | (TGA)_6_ | Cp3_F | AGCAAGGAAGACAGCGCAAG | 60.5 | 242 |
|  |  |  | Cp3_R | CGCTCCCGTCATCATCACTA | 60.5 |  |
| 13 | TRINITY_DN59_c0_g1_i4 | (GCC)_7_ | CpTest10_F | GTGTTGGTCAGCATGGAGCT | 60.5 | 290 |
|  |  |  | CpTest10_R | AAGTGTGACAGCACGACAGC | 60.5 |  |
| 14 | TRINITY_DN577_c2_g2_i2 | (TGA)_7_ | Cp4_F | ATGGACTGACGACCTGGTAG | 60.5 | 187 |
|  |  |  | Cp4_R | TGTTCCCGACCACGTTGACT | 60.5 |  |
| 15 | TRINITY_DN5668_c0_g1_i2 | (CAT)_8_ | CpTest11_F | CACCGAGCCTGTCAAAATGC | 60.5 | 292 |
|  |  |  | CpTest11_R | CAAGCGTAGTGTGCAAACACC | 61.2 |  |
| 16 | TRINITY_DN5664_c0_g1_i1 | (AT)_9_ | CpTest12_F | CCGAAAGTTAATCAACTACCTAAG | 60.3 | 206 |
|  |  |  | CpTest12_R | CCATGGCACAGTAGACAAGC | 60.5 |  |
| 17 | TRINITY_DN5467_c0_g1_i4 | (TA)_10_ | Cp5_F | CAATGTAATCGCTAATCCGTTAG | 59.2 | 224 |
|  |  |  | Cp5_R | TTCGCGCAACGCATCGTAAC | 60.5 |  |
| 18 | TRINITY_DN50349_c0_g1_i1 | (TGA)_6_ | CpTest13_F | TGGCGAACAACTAGAGAGGC | 60.5 | 192 |
|  |  |  | CpTest13_R | TACAACTGACCGGCATTCCC | 60.5 |  |
| 19 | TRINITY_DN4760_c0_g1_i19 | (CGG)_12_ | CpTest14_F | CGAGTAAGTCTAGAGACGGTC | 61.2 | 97 |
|  |  |  | CpTest14_R | CGCTCCTGTATCGTGTATCG | 60.5 |  |
| 20 | TRINITY_DN4596_c0_g1_i5 | (TA)_7_ | CpTest15_F | TGGGACGGAAATCAGACACG | 60.5 | 365 |
|  |  |  | CpTest15_R | GCGCTTTGGGTGTTGAGGTT | 60.5 |  |
| 21 | TRINITY_DN44733_c0_g1_i1 | (CT)_6_ | CpTest16_F | CGGAGTGACTATCCTGACTAC | 61.2 | 230 |
|  |  |  | CpTest16_R | CAATGTGCAGCCAAATTGGTATC | 60.9 |  |
| 22 | TRINITY_DN43616_c0_g1_i1 | (AT)_8_ | Cp6_F | CTCCAAATGGGCAAACGGAG | 60.5 | 295 |
|  |  |  | Cp6_R | ATGGAGGCTTCCTCGCTACA | 60.5 |  |
| 23 | TRINITY_DN39432_c0_g1_i1 | (CT)_6_ | CpTest17_F | TTTGACCGTCGATCAGCTGC | 60.5 | 187 |
|  |  |  | CpTest17_R | CGCCAATATCTGCCGGTCTA | 60.5 |  |
| 24 | TRINITY_DN3562_c0_g1_i9 | (TCA)_6_ | CpTest18_F | GTGGACTGCTAGTGCTGTAAC | 61.2 | 335 |
|  |  |  | CpTest18_R | ACGGTCGTACGCGCTTGTTA | 60.5 |  |
| 25 | TRINITY_DN3330_c2_g1_i1 | (TAA)_5_ | CpTest19_F | TCGATGTCGTGTTACCGGAG | 60.5 | 139 |
|  |  |  | CpTest19_R | TAGTTTGGGGCTAGATGGCG | 60.5 |  |
| 26 | TRINITY_DN32385_c0_g1_i1 | (TCA)_5_ | CpTest20_F | GGCTAGAGTAAGTGTCCAGTG | 61.2 | 182 |
|  |  |  | CpTest20_R | GTTTGCCCAAAGTGGCGAAC | 60.5 |  |
| 27 | TRINITY_DN2895_c1_g2_i2 | (TGA)7 | CpTest21_F | CCTATGTCCAGCAGTGGACT | 60.5 | 166 |
|  |  |  | CpTest21_R | GCCGCTACATAGTCCTATTGAT | 60.1 |  |
| 28 | TRINITY_DN2794_c1_g1_i3 | (CA)8 | CpTest22_F | CGACGATATGGAGTTCAGGTC | 61.2 | 237 |
|  |  |  | CpTest22_R | ACTTGAGCGCGAAATCCACG | 60.5 |  |
| 29 | TRINITY_DN2704_c0_g2_i5 | (GAA)5 | Cp7_F | ACCAACTGAGCCCTTACAGC | 60.5 | 238 |
|  |  |  | Cp7_R | AGACTCGTCACTATGACCGC | 60.5 |  |
| 30 | TRINITY_DN26105_c0_g1_i1 | (CA)13 | CpTest23_F | GCTTGTCACTTCTGTAAGGGC | 61.2 | 149 |
|  |  |  | CpTest23_R | ATGTATGTGATTATGAGTGTACC | 57.6 |  |
| 31 | TRINITY_DN2345_c0_g1_i6 | (GA)7 | CpTest24_F | GGAGCATTCTCTACCAGTTAAC | 60.1 | 196 |
|  |  |  | CpTest24_R | TTTAACGTCAGACGAGCTGTAC | 60.1 |  |
| 32 | TRINITY_DN22974_c0_g1_i1 | (CA)7 | CpTest25_F | CGGTACAGGCCAGGTTTGTA | 60.5 | 174 |
|  |  |  | CpTest25_R | GGTACTTTTTGCCCACGACTG | 61.2 |  |
| 33 | TRINITY_DN2249_c1_g1_i12 | (GAT)6 | Cp8_F | TGCGCAAGAAGGAGAAGGAG | 60.5 | 255 |
|  |  |  | Cp8_R | CCCTAGTTCGTCGGGTGTAT | 60.5 |  |
| 34 | TRINITY_DN2183_c1_g2_i1 | (ATC)5 | Cp9_F | GAACCCTCGAGCCTTGTTTC | 60.5 | 127 |
|  |  |  | Cp9_R | TGTGCCGACCCCACTTAGAG | 62.5 |  |
| 35 | TRINITY_DN2086_c0_g1_i7 | (AT)6 | CpTest26_F | AACATCTCGTACCTTTACGGTG | 60.1 | 257 |
|  |  |  | CpTest26_R | TTCAATAAACTCCCTGTGACCC | 60.1 |  |
| 36 | TRINITY_DN20246_c0_g1_i11 | (TGT)8 | CpTest27_F | CCTGCACTTGCTGCTGTATC | 60.5 | 206 |
|  |  |  | CpTest27_R | GCAAGACAAGGACCAGCAAC | 60.5 |  |
| 37 | TRINITY_DN19917_c0_g2_i1 | (TGTC)6 | CpTest28_F | CACCAGGAGCATCACATCAC | 60.5 | 148 |
|  |  |  | CpTest28_R | TTCGGAATAAACTGGCTGTGAC | 60.1 |  |
| 38 | TRINITY_DN19424_c0_g1_i2 | (TA)9 | Cp10_F | ATCCGCCTGCTTCTTGAGTG | 60.5 | 265 |
|  |  |  | Cp10_R | CCTACGAGCATCTGACCAGA | 60.5 |  |
| 39 | TRINITY_DN1842_c2_g1_i4 | (GA)7(AG)6 | CpTest29_F | TGAAGCGGAGTGGAGAAGAG | 60.5 | 274 |
|  |  |  | CpTest29_R | CGCATTCCTTTAACGGCCAG | 60.5 |  |
| 40 | TRINITY_DN1623_c0_g1_i6 | (TCA)5 | CpTest30_F | CGTCTTCATCGTCATCGTCG | 60.5 | 354 |
|  |  |  | CpTest30_R | CTGGTATCAAGCATCGAGAGC | 61.2 |  |

**Supplementary Table S3.** Hardy-Weinberg equilibrium test P-values for each microsatellite locus and population.

| **Microsatellite** | **SW** | **GJ** | **SJ** | **DG** | **CD** | **SC** | **GR** | **CJ** | **GY** | **HY** | **JP** | **PJ** | **SuC** |
| --- | --- | --- | --- | --- | --- | --- | --- | --- | --- | --- | --- | --- | --- |
| Cp1 | 0.0248^E^ | **0.0102^E^** | 0.3392^E^ | 0.4018^E^ | 0.2959^E^ | **0.0086^E^** | 0.2035^E^ | 0.2089^D^ | **0.0073^D^** | 0.6263^E^ | 0.4153^E^ | **<0.0001^E^** | **0.0001^E^** |
| Cp2 | **0.0043^E^** | **0.0001^E^** | 0.0346^E^ | 0.5511^D^ | 0.2182^E^ | **<0.0001^E^** | 0.1339^D^ | **0.0172^D^** | **0.0042^D^** | **0.0152^D^** | 0.1137^E^ | **<0.0001^E^** | **<0.0001^D^** |
| Cp3 | **<0.0001^E^** | **0.0028^E^** | 0.0282^E^ | 0.8824^E^ | 0.1922^=^ | 0.0309^E^ | 0.0889^E^ | 0.2369^D^ | 0.0435^E^ | **0.0040^D^** | 0.1964^E^ | **<0.0001^E^** | 0.0989^D^ |
| Cp4 | **0.0070^E^** | 0.1815^E^ | 0.1357^E^ | 0.2066^E^ | 0.8451^E^ | 0.0538^E^ | 0.4560^E^ | 0.1706^D^ | **0.0001^D^** | **0.0016^D^** | 0.0474^D^ | **<0.0001^D^** | **0.0088^D^** |
| Cp5 | 0.3507^E^ | **0.0010^E^** | 0.9284^E^ | 0.4390^E^ | 0.1712^E^ | **0.0212^E^** | 0.0813^E^ | **<0.0001^D^** | **0.0012^D^** | **0.0003^E^** | 0.0478^D^ | **0.0030^D^** | **<0.0001^D^** |
| Cp6 | **0.0007^E^** | **0.0036^E^** | **0.0007^E^** | **0.0026^E^** | 0.1829^E^ | **<0.0001^E^** | 0.1834^E^ | 0.5390^E^ | **<0.0001^E^** | **<0.0001^E^** | 0.2280^E^ | **<0.0001^E^** | 0.5734^E^ |
| Cp7 | 0.1025^E^ | 0.2749^E^ | 0.8070^E^ | 0.2742^E^ | 0.7835^E^ | 0.2160^E^ | 0.6150^E^ | 0.1063^D^ | 0.3957^D^ | **0.0034^D^** | 0.3107^D^ | **<0.0001^D^** | 0.2534^D^ |
| Cp8 | 0.0258^E^ | **<0.0001^E^** | **0.0007^E^** | 0.2894^E^ | 0.9142^E^ | **<0.0001^E^** | 0.2376^E^ | 0.1528^D^ | **0.0009^D^** | **0.0002^D^** | 0.0297^D^ | **<0.0001^E^** | 0.1549^D^ |
| Cp9 | 0.5620^E^ | 0.3290^E^ | 0.1798^E^ | 0.9307^E^ | 0.6270^D^ | **0.0149^E^** | 0.6065^E^ | **0.0001^D^** | 0.0442^D^ | **0.0097^D^** | 0.2698^E^ | **<0.0001^D^** | 0.0253^E^ |
| Cp10 | **0.0218^E^** | 0.3623^E^ | **0.0204^E^** | 0.1110^E^ | 0.5680^E^ | 0.5356^E^ | 0.1424^E^ | **<0.0001^D^** | **<0.0001^D^** | **<0.0001^D^** | **0.0033^D^** | **<0.0001^E^** | **<0.0001^D^** |

P-values < 0.05 indicate significant deviation from Hardy-Weinberg equilibrium. Values shown as <0.0001 correspond to values reported as 0.0000 in the source table. Values are unadjusted exact-test P-values. Bold values remained significant at q<0.05 after Benjamini–Hochberg false-discovery-rate correction across the 130 tests. Superscript D indicates heterozygote deficiency, E indicates heterozygote excess, and = indicates equal observed and expected heterozygosity.

**Supplementary Table S4.** FreeNA estimates of null allele frequencies and comparison of F_ST_ values with and without ENA correction in *C. punctiferalis*.

| **Locus information** | **Null allele frequency estimates** | | | | | **Effect of ENA correction on F_ST_** | | |
| --- | --- | --- | --- | --- | --- | --- | --- | --- |
|  | **Mean r** | **Maximum r** | **Population at maximum r** | **No. populations with r > 0.10** | **No. populations with r > 0.20** | **F_ST_ without ENA** | **F_ST_ with ENA** | **Change (%)** |
| Cp1 | 0.009 | 0.121 | GY | 1 | 0 | 0.1030 | 0.0971 | 5.8 |
| Cp2 | 0.029 | 0.105 | GR | 1 | 0 | 0.1402 | 0.1352 | 3.5 |
| Cp3 | 0.021 | 0.168 | HY | 1 | 0 | 0.0623 | 0.0570 | 8.4 |
| Cp4 | 0.053 | 0.267 | PJ | 3 | 1 | 0.0519 | 0.0492 | 5.2 |
| Cp5 | 0.049 | 0.193 | SuC | 4 | 0 | 0.1100 | 0.0948 | 13.8 |
| Cp6 | 0.002 | 0.020 | SJ | 0 | 0 | 0.1090 | 0.1093 | -0.2 |
| Cp7 | 0.048 | 0.281 | PJ | 3 | 1 | 0.1459 | 0.1333 | 8.6 |
| Cp8 | 0.029 | 0.097 | JP | 0 | 0 | 0.0934 | 0.0877 | 6.1 |
| Cp9 | 0.043 | 0.259 | PJ | 2 | 1 | 0.0890 | 0.0836 | 6.0 |
| Cp10 | 0.068 | 0.264 | GY | 4 | 3 | 0.1208 | 0.1075 | 11.0 |
| Global F_ST_ (all loci) | | | | | | 0.1019 | 0.0951 | 6.7 |

Notes: *r* indicates the null allele frequency estimated by FreeNA; ENA indicates the excluding null alleles (ENA) correction. F_ST_ values are Weir’s F_ST_ estimates. Positive change values indicate a decrease after ENA correction. Global F_ST_ 95% CIs: without ENA, 0.1019 (0.0845–0.1199); with ENA, 0.0951 (0.0783–0.1119).

**Supplementary Table S5.** Pairwise genotypic linkage disequilibrium tests among ten microsatellite loci in 13 populations of *C. punctiferalis.*

| **Locus pair** | **PJ** | **SW** | **CJ** | **SJ** | **GJ** | **DG** | **CD** | **HY** | **SC** | **GR** | **GY** | **SuC** | **JP** |
| --- | --- | --- | --- | --- | --- | --- | --- | --- | --- | --- | --- | --- | --- |
| Cp1–Cp2 | 0.7161 | 0.7375 | 0.0829 | 1.0000 | 0.7072 | 0.2218 | 1.0000 | **<0.0001** | 0.9747 | 1.0000 | 0.5979 | 0.1834 | 0.1454 |
| Cp1–Cp3 | 0.1405 | 0.7333 | **0.0003** | 0.4744 | 0.9132 | 1.0000 | 1.0000 | 0.0147 | 0.9836 | 0.0491 | 0.5294 | 0.0039 | 0.9699 |
| Cp2–Cp3 | 0.0783 | 0.7050 | **0.0001** | 0.5510 | 0.0933 | 0.2392 | 1.0000 | 0.0054 | 0.9787 | 0.7900 | 0.1034 | 0.0329 | 0.2118 |
| Cp1–Cp4 | 0.1605 | 0.0780 | 0.3499 | 0.1793 | 0.4144 | 1.0000 | 0.4500 | 0.1902 | 0.4484 | 1.0000 | 0.0101 | 0.1490 | 0.1646 |
| Cp2–Cp4 | 0.1040 | 0.5733 | 0.9674 | 1.0000 | 0.2259 | 1.0000 | 1.0000 | 0.0698 | 0.4523 | 0.7237 | 0.3248 | 0.1458 | 0.3063 |
| Cp3–Cp4 | 0.6949 | 0.8675 | 0.9381 | 0.6071 | 0.4331 | 1.0000 | 0.3743 | 0.0388 | 0.4979 | 0.9090 | 0.5775 | 0.0040 | 0.2649 |
| Cp1–Cp5 | 0.4455 | 0.3759 | 0.0116 | 0.5868 | 0.3514 | 0.6722 | 1.0000 | 0.0194 | 0.1783 | 0.2742 | 0.0046 | 0.1345 | 0.1756 |
| Cp2–Cp5 | 0.3651 | 0.1004 | **<0.0001** | 0.6377 | 0.1081 | 0.0254 | 0.4777 | 0.0287 | 0.7341 | 0.2341 | 0.2125 | 0.0147 | 0.1777 |
| Cp3–Cp5 | 0.0388 | 0.1577 | 0.0333 | 0.5843 | 0.4591 | 1.0000 | 1.0000 | 0.0535 | 0.9877 | 0.8247 | 0.1004 | 0.1576 | 0.0844 |
| Cp4–Cp5 | 0.4175 | 0.9878 | 0.9708 | 0.7191 | 0.2795 | 0.6858 | 1.0000 | 0.4499 | 0.8110 | 0.7798 | 0.1161 | 0.1496 | 0.4617 |
| Cp1–Cp6 | 0.7266 | 0.6859 | 0.0469 | 0.5675 | 0.2065 | 0.8695 | 1.0000 | **<0.0001** | 0.3065 | 0.2345 | **<0.0001** | 0.0149 | 0.0857 |
| Cp2–Cp6 | 0.0137 | 0.7258 | **0.0012** | 0.7005 | 1.0000 | 0.6136 | 1.0000 | **<0.0001** | 0.7089 | 0.5439 | 0.1338 | 0.0035 | 0.1303 |
| Cp3–Cp6 | 0.2567 | 0.0872 | 0.0156 | 0.4990 | 0.9342 | 1.0000 | 1.0000 | **0.0027** | 0.2608 | 0.4195 | 0.4425 | **<0.0001** | 0.2264 |
| Cp4–Cp6 | 0.0860 | 0.1276 | 0.8748 | 0.9640 | 0.7491 | 0.2154 | 1.0000 | 0.0386 | 0.6009 | 0.8728 | 0.1184 | 0.1369 | 0.1025 |
| Cp5–Cp6 | 0.1969 | 0.6546 | 0.0140 | 0.7296 | 0.6957 | 0.4405 | 0.7477 | **0.0022** | 0.1521 | 0.5666 | 0.1038 | **0.0010** | 0.0111 |
| Cp1–Cp7 | 0.1546 | 0.9716 | 0.8151 | 0.6280 | 0.9250 | 0.8177 | 1.0000 | 0.3337 | 0.3909 | 0.0371 | 0.5117 | 0.6551 | 0.7081 |
| Cp2–Cp7 | 1.0000 | 0.1269 | 0.3149 | 0.0781 | 0.3362 | 0.4818 | 0.2369 | 0.0491 | 0.1473 | 0.9129 | 0.5521 | 0.2585 | 0.3530 |
| Cp3–Cp7 | 0.8108 | 0.0695 | 0.7074 | 0.5865 | 1.0000 | 0.0205 | 1.0000 | 0.1125 | 0.6035 | 0.8080 | 0.7193 | 0.9792 | 0.0527 |
| Cp4–Cp7 | 0.0445 | 0.8855 | 0.9099 | 0.1441 | 0.1703 | 0.1510 | 1.0000 | 0.3718 | 0.7499 | 0.7932 | 0.8991 | 0.5385 | 0.8988 |
| Cp5–Cp7 | 1.0000 | 0.2469 | 0.6904 | 0.0973 | 0.5409 | 0.8515 | 0.1010 | 0.1343 | 0.0912 | 0.9264 | 0.9867 | 0.0786 | 0.3829 |
| Cp6–Cp7 | 1.0000 | 0.5464 | 0.5838 | 0.7478 | 0.7231 | 0.9651 | 1.0000 | 0.2767 | 0.8325 | 0.5442 | 0.2881 | 0.4062 | 0.6423 |
| Cp1–Cp8 | 0.8146 | 0.0779 | 0.0795 | 0.7796 | 0.3738 | 1.0000 | 1.0000 | 0.1284 | 0.5359 | 0.0508 | 0.2091 | 0.0286 | 0.2616 |
| Cp2–Cp8 | 0.0326 | 0.9352 | 0.4043 | 0.8220 | 0.2312 | 0.5936 | 0.1395 | 0.2441 | 0.8737 | 0.8060 | **0.0026** | **0.0009** | 0.1155 |
| Cp3–Cp8 | 0.0152 | 0.9528 | 0.4849 | 0.7657 | 0.8639 | 1.0000 | 1.0000 | 0.1595 | 0.4205 | 0.6500 | 0.0152 | **<0.0001** | 0.6875 |
| Cp4–Cp8 | 0.2646 | 0.3045 | 0.4090 | 0.5117 | 0.4930 | 0.0816 | 1.0000 | **0.0023** | 0.3817 | 1.0000 | 0.3222 | 0.0403 | 0.7539 |
| Cp5–Cp8 | 0.0122 | 0.4681 | 0.0389 | 0.8539 | 0.3228 | 0.5982 | 0.3364 | **<0.0001** | 0.2508 | 0.0245 | 0.1526 | **<0.0001** | 0.1981 |
| Cp6–Cp8 | 0.4193 | 0.6060 | 0.6241 | 0.1392 | 0.3558 | 1.0000 | 1.0000 | **0.0006** | 0.7278 | 0.1859 | 0.2289 | **<0.0001** | 0.4166 |
| Cp7–Cp8 | 0.7979 | 0.8767 | 0.9999 | 0.2415 | 0.4738 | 0.7032 | 0.1816 | 0.0756 | 0.9771 | 0.4772 | 0.2055 | 0.5625 | 0.7178 |
| Cp1–Cp9 | 0.0268 | 0.2002 | 0.0949 | 0.3050 | 0.6939 | 1.0000 | 1.0000 | 0.4804 | 0.9880 | 0.1997 | 0.2210 | 0.1510 | 0.4311 |
| Cp2–Cp9 | 0.7504 | 0.1819 | 0.4175 | 1.0000 | 0.2588 | 0.6984 | 1.0000 | 0.4176 | 0.1292 | 0.8710 | 0.4677 | **0.0028** | 0.4210 |
| Cp3–Cp9 | 0.0403 | 0.5394 | 0.4286 | 0.2588 | 0.6252 | 0.2009 | 0.1252 | 0.1915 | 0.0145 | 0.1374 | 0.1872 | **0.0021** | 0.5940 |
| Cp4–Cp9 | 0.1537 | 0.1165 | 0.8430 | 0.4500 | 0.2868 | 0.6012 | 1.0000 | 0.4268 | 0.1082 | 0.3532 | 0.6266 | 0.1299 | 0.5903 |
| Cp5–Cp9 | 0.0178 | 0.2251 | 0.5302 | 0.4108 | 0.8339 | 0.6860 | 1.0000 | 0.6220 | 0.1461 | 0.2810 | 0.0394 | 0.1733 | 0.6683 |
| Cp6–Cp9 | 0.4206 | 0.4552 | 0.7234 | 0.6345 | 0.9467 | 0.2539 | 1.0000 | 0.2161 | 0.6894 | 0.3419 | 0.3836 | 0.0094 | 0.2447 |
| Cp7–Cp9 | 0.2748 | 0.2068 | 0.4573 | 0.1271 | 0.6382 | 0.3343 | 0.1642 | 0.6849 | 0.1427 | 0.6968 | 0.3289 | 0.1173 | 0.0834 |
| Cp8–Cp9 | 0.0350 | 0.9754 | 0.4913 | 0.7200 | 1.0000 | 0.0060 | 1.0000 | 0.7671 | 0.0161 | 0.2230 | 0.4404 | 0.0047 | 0.5795 |
| Cp1–Cp10 | **<0.0001** | 0.2632 | 0.0267 | 0.8726 | 1.0000 | 0.0342 | 1.0000 | 0.0036 | 0.4114 | 0.2735 | **0.0029** | **0.0029** | 0.0054 |
| Cp2–Cp10 | 1.0000 | 0.8722 | **0.0028** | 0.2287 | 0.3121 | 0.0503 | 1.0000 | 0.0322 | 0.8383 | 1.0000 | 0.0089 | 0.0059 | 0.0232 |
| Cp3–Cp10 | 0.3485 | 1.0000 | 0.0173 | 0.4067 | 0.5410 | 1.0000 | 1.0000 | **0.0009** | 1.0000 | 1.0000 | 0.2884 | **<0.0001** | 0.7884 |
| Cp4–Cp10 | 0.2174 | 0.2404 | 0.8643 | 0.9401 | 0.8395 | 1.0000 | 0.3812 | 0.1058 | 0.0582 | 1.0000 | 0.3457 | 0.0151 | 0.8453 |
| Cp5–Cp10 | 1.0000 | 0.3622 | 0.1322 | 0.9298 | 0.0354 | 0.8409 | 1.0000 | 0.0180 | 0.3387 | 0.0592 | 0.0031 | **<0.0001** | 0.0433 |
| Cp6–Cp10 | 1.0000 | 0.2442 | **0.0003** | **0.0016** | 0.7019 | 0.6803 | 1.0000 | **<0.0001** | 0.5971 | 1.0000 | 0.0531 | **<0.0001** | 0.0251 |
| Cp7–Cp10 | 0.1480 | 0.7612 | 0.9743 | 0.7108 | 0.4961 | 0.9186 | 1.0000 | 0.3512 | 0.7015 | 1.0000 | 0.9135 | 0.5084 | 0.7544 |
| Cp8–Cp10 | 1.0000 | 0.0690 | **<0.0001** | 0.1910 | 0.4826 | 0.6633 | 1.0000 | 0.0147 | 0.5766 | 0.7168 | 0.0367 | **<0.0001** | 0.2355 |
| Cp9–Cp10 | 0.0076 | 0.7760 | 0.5092 | 0.9893 | 0.1680 | 0.7732 | 1.0000 | 0.0755 | 0.9301 | 0.8035 | 0.0390 | 0.0643 | 0.6158 |

Notes: Values are unadjusted exact-test P-values obtained in GENEPOP v. 4.8.6 using 10,000 dememorization steps, 100 batches, and 5,000 iterations per batch. Bold values remained significant at q < 0.05 after Benjamini–Hochberg false-discovery-rate correction across the 585 tests.

**Supplementary Table S6.** Relative directional connectivity matrix based on Jost's D estimated by divMigrate after excluding the Japanese population. Rows indicate source populations, and columns indicate recipient populations.

| **Source / Recipient** | **SW** | **GJ** | **SJ** | **DG** | **CD** | **SC** | **GR** | **CJ** | **GY** | **HY** | **PJ** | **SuC** |
| --- | --- | --- | --- | --- | --- | --- | --- | --- | --- | --- | --- | --- |
| **SW** | - | 0.115 | 0.693 | 0.106 | 0.442 | 0.082 | 0.101 | 0.105 | 0.054 | 0.063 | 0.047 | 0.067 |
| **GJ** | 0.077 | - | 0.087 | 0.023 | 0.039 | 0.563 | 0.024 | 0.025 | 0.017 | 0.022 | 0.015 | 0.015 |
| **SJ** | 0.391 | 0.171 | - | 0.119 | 0.175 | 0.116 | 0.099 | 0.109 | 0.056 | 0.066 | 0.045 | 0.055 |
| **DG** | 0.131 | **0.166** | 0.207 | - | 0.125 | **0.170** | 0.703 | 0.105 | 0.080 | 0.087 | 0.051 | 0.075 |
| **CD** | 0.830 | **0.223** | 0.458 | 0.149 | - | **0.121** | 0.129 | 0.103 | 0.058 | 0.081 | 0.052 | 0.076 |
| **SC** | 0.064 | 0.606 | 0.058 | 0.020 | 0.033 | - | 0.021 | 0.020 | 0.015 | 0.018 | 0.012 | 0.013 |
| **GR** | 0.180 | **0.142** | 0.225 | 1.000 | 0.134 | **0.179** | - | 0.189 | 0.146 | 0.113 | 0.066 | 0.117 |
| **CJ** | **0.427** | **0.225** | 0.373 | 0.233 | 0.354 | **0.315** | 0.413 | - | 0.230 | 0.388 | 0.176 | 0.306 |
| **GY** | **0.182** | **0.153** | **0.229** | 0.260 | 0.188 | **0.171** | 0.498 | 0.427 | - | 0.437 | 0.172 | 0.796 |
| **HY** | **0.210** | **0.121** | **0.257** | 0.194 | 0.259 | **0.174** | 0.226 | 0.303 | 0.263 | - | 0.094 | 0.210 |
| **PJ** | **0.374** | **0.199** | **0.354** | **0.342** | **0.366** | **0.399** | **0.576** | 0.476 | 0.226 | 0.294 | - | 0.258 |
| **SuC** | **0.214** | **0.157** | **0.246** | 0.329 | 0.221 | **0.122** | 0.552 | 0.609 | 0.649 | 0.313 | 0.163 | - |

Note: JP was excluded from this analysis. Values are rounded to three decimal places and were normalized so that the maximum value equaled 1.0. Bold values indicate the larger direction in reciprocal comparisons for which the 95% bootstrap confidence intervals did not overlap, based on 1,000 bootstrap replicates. Unbolded values are point estimates and do not indicate bootstrap-supported directional asymmetry.
